# Ascorbate: a forgotten component in the cytotoxicity of Cu(II) ATCUN peptide complexes

**DOI:** 10.1007/s00775-024-02083-9

**Published:** 2024-11-11

**Authors:** Julian Heinrich, Elisa Siddiqui, Henrike Eckstein, Michael Naumann, Nora Kulak

**Affiliations:** 1 Institute of Chemistry, University of Potsdam, Karl-Liebknecht-Straße 24-25, 14476 Potsdam, Germany; 2 Institute of Chemistry, Otto von Guericke University, Universitätsplatz 2, 39106 Magdeburg, Germany; 3 Institute of Experimental Internal Medicine, Otto von Guericke University, Leipziger Straße 44, 39120 Magdeburg, Germany

**Keywords:** Copper(II) complex, ATCUN peptide, Cytotoxicity, Ascorbate

## Abstract

In 1983, Linus Pauling and colleagues reported about enhanced antitumor activity of the Cu(II) complex of the simplest ATCUN (amino terminal Cu(II) and Ni(II)-binding motif) peptide (NH_2_-Gly-Gly-His-COOH, GGH) in the presence of ascorbate as an additive. In the following 4 decades, structural modifications of this complex were implemented, however, anticancer activity could not be significantly increased. This has led to neglecting the ATCUN motif and its Cu(II) complexes as potential chemotherapeutic agents. Furthermore, the addition of ascorbate with its positive effect on the anticancer activity has fallen into oblivion. In this work, we compared Cu(II) GGH with Cu(II) ATCUN peptides bearing β-Ala instead of Gly at the 2nd position of the peptide sequence regarding their in vitro complex stability and cytotoxicity (MTT (3-(4,5-dimethylthiazol-2-yl)-2,5-diphenyltetrazolium bromide) and annexin V-FITC (fluorescein isothiocyanate) apoptosis assay) towards three cancer cell lines (AGS, HeLa and NCI-N87). Such an exchange of amino acids led to an up to three-fold higher cytotoxic effect in the presence of ascorbate. We thus achieved a significant increase in the otherwise moderate cytotoxicity of Cu(II) ATCUN-like complexes. Lipophilicity assays (*n*-octanol/water coefficient, log *P* values) of the studied complexes were used to evaluate differences in the antiproliferative activity.

**Graphical abstract:**

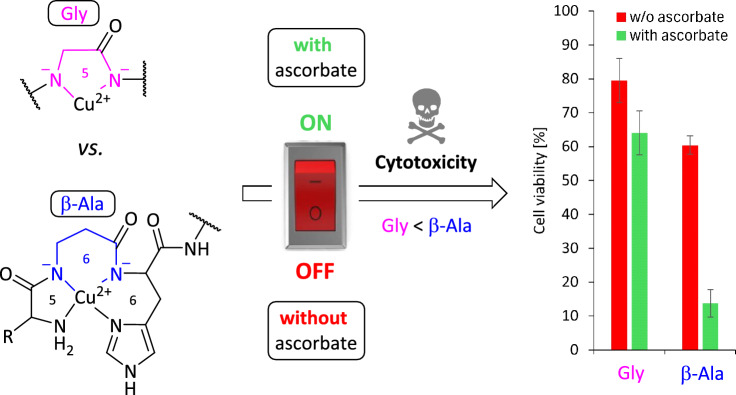

**Supplementary Information:**

The online version contains supplementary material available at 10.1007/s00775-024-02083-9.

## Introduction

Due to its biological importance, deoxyribonucleic acid (DNA) is a promising and thus common target to induce apoptosis in malignant cells, e.g. in the treatment of cancer [1]. One of the most successfully used chemotherapeutic agents is cisplatin. Within this metal complex the Pt(II) cations produces irreversible DNA crosslinkings which consequently lead to cell death. Disadvantages of the clinical application of cisplatin are severe side effects, such as nephro- and neurotoxicity [2]. To overcome these side effects, current research is also focused on metal complexes based on endogenous metals like Cu for a potential usage as anticancer agents accompanied with a lower systemic toxicity [3, 4].

**Fig. 1:**
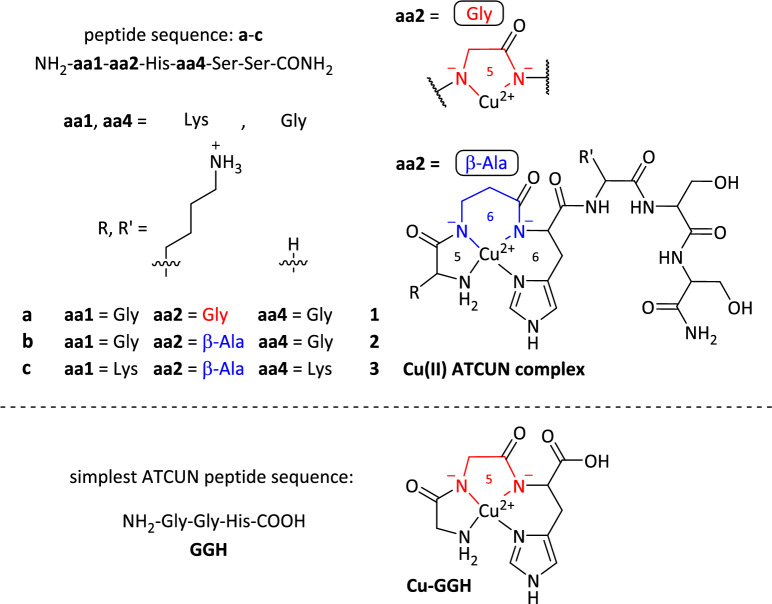
Cu(II) complexes **1**–**3** of ATCUN peptides **a**-**c**. Amino acid **aa2** is either Gly (α-amino acid, 5-membered chelate) or β-Ala (β-amino acid, 6-membered chelate), whereas **aa1** and **aa4** are either Gly or Lys. **Cu-GGH** is the Cu(II) ATCUN complex as synthesized from Linus Pauling and coworkers [10, 11]. **1**–**3** are amidated and **Cu-GGH** is carboxylated at the *C*-terminus

Tripeptides with an ATCUN (amino terminal Cu(II) and Ni(II) binding) motif are able to bind Cu(II) in a square-planar fashion (4N chelating ligand) through a N-terminal amine group, two deprotonated peptide bonds and a δ-N atom of the imidazole moiety (N_Im_) of His (Fig. 1). His as the third amino acid in the peptide sequence is essential for its Cu(II) binding and complex stability [5, 6]. The ATCUN motif occurs at the N-terminus of albumins [7], neuromedin C [8] and the Cu transport protein Ctr1 [9].

A milestone in the evaluation of the cytotoxicity of such Cu(II) ATCUN peptide complexes (metallopeptides) was achieved by Linus Pauling and coworkers in 1983. They synthesized the Cu(II) complex with the simplest ATCUN peptide (NH_2_-Gly-Gly-His-COOH = GGH) as ligand and showed that this Cu(II)-GGH complex has a high antitumor activity in the presence of ascorbate (AscH^−^) against Ehrlich ascites tumor cells in vitro and as well inoculated in mice (in vivo) [10]. In the same year, at the Linus Pauling Institute of Science and Medicine, an oxidative DNA-damage induced by reactive oxygen species (ROS) production was proven by this Cu(II)-GGH-ascorbate system in presence of O_2_ [11].

**Scheme 1:**
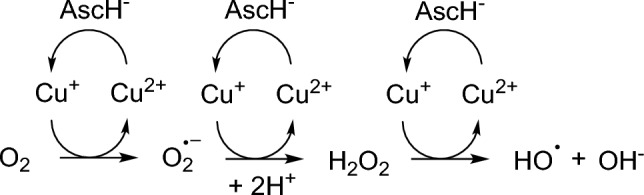
Commonly accepted ROS generation for Cu(II) complexes. AscH^−^ initiates the Cu(II)/Cu(I) redox cycling for ROS evolution [5, 19]

From 1983 on novel peptides were designed by modifying the simplest Cu(II)-GGH complex to improve the anticancer and nuclease activity. Foremost, Cowan et al*.* reported on a variety of Cu(II) ATCUN complexes with several biological activities (anticancer, -viral, and -microbial, DNA cleavage and enzyme inhibition) [1, 12, 13]. With respect to the anticancer properties of such metallopeptides, complexes with rather similar and/or attenuated activities were gained in comparison to Cu(II)-GGH [14, 15]. This fact is mainly caused by the high complex stability at pH 7.4 (log *K*_7.4_ = 12.4). The 4N-donor peptide GGH forms stable chelate rings (5,5,6) within the complex structure (Fig. 1) [5]. Hence, the Cu(II) is”fixed” in a square-planar complex geometry due to (modified) GGH. The Cu(II) complex is unable to switch to a tetrahedral Cu(I) geometry, which is the key point to initiate ROS production (Scheme 1) [5, 16]. Additionally, with respect to the antitumor activity evaluation of newly synthesized Cu(II) ATCUN complexes in the last 40 years, the usage of AscH− as additive has fallen into oblivion in in vitro cytotoxicity experiments [14, 15]. This might be due to Linus Pauling’s activities in the context of vitamin C for the prevention of cancer, which have been criticized a lot [17].

In general, these facts lead to neglecting the ATCUN motif and its Cu(II) complexes as promising ROS-generating candidates for an application in chemotherapy. Recently, our group reported that an exchange of the second amino acid Gly (Gly2) for β-Ala (β-Ala2) from the N-terminus in complexes of the type Cu(II)-GGH increases the overall biological activity due to a higher flexibility in the ligand scaffold (5,5,6 *vs.* 5,6,6 chelates) (Fig. 1). We showed that the tetrahedral Cu(I) geometry is more accessible, which results in an enhanced ROS production and DNA cleavage in vitro and significant higher cytotoxicity towards cancer cells [18].

In this work, on selected Cu(II) ATCUN complexes (Fig. 1), we investigate the usage of AscH^−^ as additive in cytotoxicity experiments. Various cancer cell lines were treated and different methods for cytotoxicity evaluation were applied.

## Materials and methods

All chemicals and solvents were purchased from *Sigma-Aldrich*, *Essen BioScience*, *Fisher Scientific* and *Life Technologies*, respectively, and were used without further purification. Copper(II) chloride (CuCl_2_) was used as the dihydrate. As AscH^−^ source an aqueous sodium ascorbate (sodium salt of vitamin C) solution was used. Milli-Q® water (18 MΩ·cm) was used as a solvent if not stated otherwise. HeLa (CCL-2), AGS (CRL-1739) and NCI-N87 (CRL-5822) cancer cells were supplied by *ATCC GmbH*. UV/VIS experiments were carried out on an *Agilent Cary 100 Bio UV/VIS spectrophotometer*.

### Cu(II) complex preparation and stability

The Cu(II) ATCUN complexes **1**–**3** and **Cu-GGH** were prepared in situ as described before [18]. Here, in all experiments, the corresponding complex was used in a Cu(II):peptide ratio of 1:1.05 in order to prevent non-coordinated Cu(II) from reacting.

For the evaluation of complex stability of **1**–**3** and **Cu-GGH** under physiologically conditions, an aqueous 1 mM complex solution in 50 mM HEPES buffer (pH 7.4) at 37 °C was followed for 48 h by UV/VIS spectroscopy utilizing λ_max_ of the d-d transition band of each corresponding complex. λ_max_ is 525 nm for **1** and **Cu-GGH**, and 545 nm for **2** and **3**.

### Cytotoxicity studies

The complexes **1**–**3**, **Cu-GGH**, CuCl_2_ and the peptides **a**-**c**, **GGH** alone were tested on three different cancer cell lines towards their cytotoxic activity: HeLa cells (cervical adenocarcinoma), AGS and NCI-N87 cells (both gastric adenocarcinoma). The cells were grown and treated with compounds in RPMI Medium 1640 (1×) supplemented with 10% fetal calf serum in a humidified incubator with 5% CO_2_ at 37 °C. MTT cell viability assays were carried out at two concentrations (100 and 250 µM) at least in duplicate (in AGS cells additionally in two independent experiments). The Annexin V-FITC apoptosis assay was carried out at a compound concentration of 500 µM in duplicate in two independent experiments.

#### MTT cell viability assay

For the MTT cell viability assay (*Sigma Aldrich*), cells were seeded at 10 000 (HeLa and AGS) and 20 000 (NCI-N87) cells/well in 96-well plates and left to settle for 24 h. After the media was changed, **1**–**3**, **Cu-GGH** and CuCl_2_ (100 and 250 µM) and the peptides **a**-**c**, **GGH** (105 and 262.5 µM) were added with or without AscH^−^ (100 or 0 µM) as aqueous solutions for 48 h (incubation time) before the assay was started by addition of MTT (end concentration of 0.5 mg/mL). Cell metabolization of MTT to purple formazan crystals was carried out for 4 h incubation time. Afterwards, the cell culture medium was removed carefully before 100 µL DMSO was added to dissolve the purple formazan crystals for 30 min on a plate shaker. Quantification of the formazan was ensured by a *Spectramax M5* (*Molecular Devices*) microplate reader at an absorbance of 470 nm to determine the percentage of cell-growth inhibition of each sample (with compound) relative to a negative control (no compound). As positive control cytotoxic *N*-ethylmaleimide (NEM) was used in the assay.

#### Annexin V-FITC apoptosis assay

For the annexin V-FITC apoptosis assay (*Essen BioScience*), cells were seeded at 250 000 (HeLa and AGS) and 500 000 (NCI-N87) cells/dish in 15 × 60 mm cell culture dishes and left to settle for 24 h. After the media was changed, **1**–**3**, **Cu-GGH** and CuCl_2_ (500 µM) and the peptide **a** (525 µM), as an exemplary peptide, were added with or without AscH^−^ (100 or 0 µM) as aqueous solutions for 40 h. After incubation, the cells were processed by steps of washing, transferring and centrifugation before the corresponding cell pellets were treated with a 100 µL mixture of Annexin-V-FITC, propidium iodide (PI) and 1 × Annexin-incubation buffer (ratio of 5:5:90). The resuspended cells were incubated for 30 min in the dark. After centrifugation, the cell pellets were mixed with 200 µL 1 × Annexin-incubation buffer and analyzed through the FACS (fluorescence-assisted cell sorting) technique by a *CyFlow* (*Partec*) flow cytometer (annexin V-FITC: λ_ex_ = 488 nm, λ_em_ = 519 nm; PI: λ_ex_ = 488 nm, λ_em_ = 617 nm).

### Lipophilicity

Log *P* determination, where *P* is the water-*n*-octanol partition coefficient, was adapted from Bonnet et al. [20] and Khnayzer et al*.* [21] with a slightly different procedure protocol while taking into consideration the EPA guidelines (EPA 712–C–96–038).

To 1 mL of a 1.25 mM complex/CuCl_2_ solution in *n*-octanol-saturated water (10 mM HEPES, pH 7.4) were pipetted 1 mL water saturated *n*-octanol in a 2 mL Eppendorf tube. The Eppendorf tubes were shaken by hand every 10 min for 1 h at room temperature. The samples were centrifuged for 10 min at approx. 6000 rpm with a *FisherBrand SPROUT Mini Centrifuge*. Two phases were formed: An upper *n*-octanol and a lower water phase. The upper *n*-octanol phase was decanted off. 750 μL of the lower water phase was carefully pipetted into a cuvette. The Cu(II) complex concentration in the water phase was determined photometrically through the corresponding absorption coefficient ε at λ_max_ of the d-d transition (for **1** λ_max_ (ε) = 525 nm (83.282 L mol^−1^ cm^−1^), **2** λ_max_ (ε) = 545 nm (55.363 L mol^−1^ cm^−1^), **3** λ_max_ (ε) = 545 nm (59.777 L mol^−1^ cm^−1^), **Cu-GGH** λ_max_ (ε) = 525 nm (86.908 L mol^−1^ cm^−1^) and CuCl_2_ λ_max_ (ε) = 695 nm (26.328 L mol^−1^ cm^−1^), which was determined in an independent UV/VIS experiment (data not shown)). The experiment was carried out in duplicate, and one sample contained no complex, where the water phase was applied as baseline in the UV/VIS absorption measurement. The following equation was used to calculate the log *P* values.

$$\log P = {\text{log}}\frac{{\left[ {complex} \right]_{octanol} }}{{\left[ {complex} \right]_{aq} }} = {\text{log}}\frac{{\left[ {complex} \right]_{total} - \left[ {complex} \right]_{aq} }}{{\left[ {complex} \right]_{aq} }}$$

## Results and discussion

Recently, the Cu(II) complexes **1**–**3** and **Cu-GGH** and their corresponding peptides **a**-**c** and **GGH** were characterized thoroughly at pH 7.4 regarding their complex structures/stability, redox potential and in vitro biological activity, i.e. ROS generation and DNA cleavage/binding. It was shown that an exchange of Gly2 (here, **1** and **Cu-GGH**) in the ATCUN motif for β-Ala2 (here, **2** and **3**) lead to an overall increase in the bioactivity (Gly (5,5,6) < β-Ala (5,6,6 chelates)). This trend was also observed in preliminary cytotoxicity studies towards cancer cells, notably without AscH^−^ as additive [18]. For the present work, the cell study experiments were intensified.

### Cu(II) complex stability

**Fig. 2:**
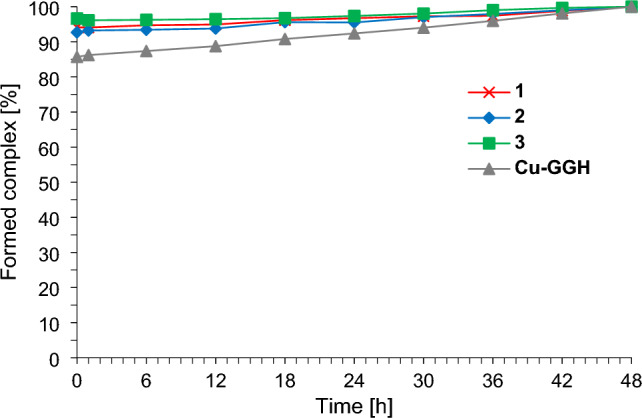
Complex stability of the compounds **1**–**3** and **Cu-GGH** within 48 h by considering the characteristic d-d transition band of the corresponding complex via UV/VIS spectroscopy (for **1** and **Cu-GGH** (Gly) λ_max_ is 525 nm, for **2** and **3** (β-Ala) 545 nm). For each complex the time point with the highest d-d band absorbance was ascribed to 100% formed complex, to which absorbances at other time points of the experiment were related. For UV/VIS spectra of the complexes see Fig. S1–S4

For the cytotoxic evaluation of such complexes, the complex stability at physiological conditions (37 °C, pH 7.4) is inevitable for at least the duration of the corresponding cytotoxicity experiment (here 48 h). For this reason, the absorbance of complexes **1**–**3** and **Cu-GGH** were monitored in solution by UV/VIS spectroscopy taking into account the characteristic d-d transition of each complex (see Fig. 2).

Based on the fact that the d-d transition band of the Cu(II) complexes **1**–**3** is not changed significantly within 48 h (see UV/VIS spectra in Fig. S1–S4 and stability plot in Fig. 2) the complexes are stable under physiological conditions in vitro (37 °C, pH 7.4). In the UV/VIS spectrum of **Cu-GGH**, however, the d-d band is 2 nm red-shifted and the absorbance is increased after 48 h of measurement (about 14%, see Fig. S4). The red-shift can be explained by intramolecular coordination of the carboxylate in the GGH peptide (see Fig. 1) in axial position leading to a square-based pyramidal coordination of Cu(II) [18, 22]. Additionally, such a perturbation of square-planar geometry of Cu(II)-4N complexes is known to result in an increase of the absorption coefficient ε [22]. It can thus be assumed that the 4N-ATCUN coordination stays intact for **Cu-GGH** as well as for complexes **1**–**3**.

### Cytotoxicity studies

**Fig. 3:**
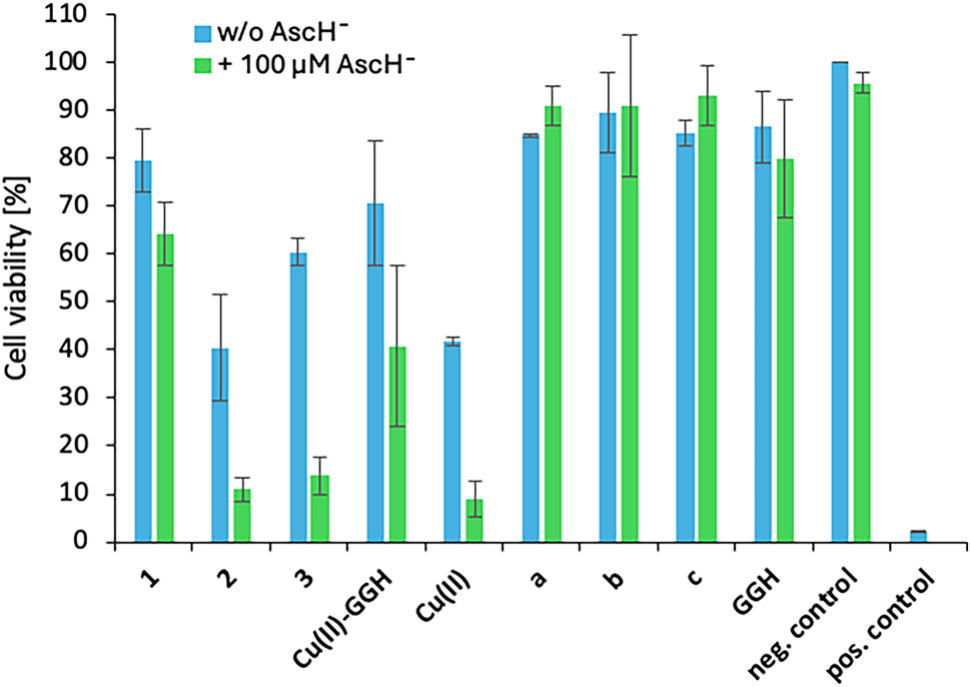
Cytotoxic effect of the complexes **1**–**3**, **Cu-GGH** (250 µM) and their corresponding peptide ligands **a**-**c**, **GGH** alone (262.5 µM) and CuCl_2_, indicated as Cu(II), (250 µM) on the cell viability of AGS cells in the absence/presence (100 µM) of AscH^−^ as additive measured by the MTT assay after 48 h treatment. Higher concentrations of the ligands were used in accordance to the Cu(II):peptide ratio in the complexes of 1:1.05

For cytotoxicity evaluation of the compounds towards cancer cells, first the MTT cell viability assay was used on AGS, NCI-N87 and HeLa cells. MTT (yellowish) is metabolized by living cells to a formazan derivative (purple-colored) in crystal form, the amount of which corresponds to the number of living cells, and hence reveal cell viability data [23]. For the sake of comparison, not only the complexes **1**–**3** and **Cu-GGH**, but also the peptide ligands **a**-**c**, **GGH** and the metal salt CuCl_2_ alone were tested. In Fig. 3, the cytotoxic effect on cell viability (MTT assay) of all compounds (250 µM complex and metal salt, 262.5 µM peptide ligand) without or with AscH^−^ (100 µM) as additive on AGS cells is shown (see Fig. S5 and S6 for HeLa and NCI-N87 cells).

All compounds were tested without or with AscH^−^ (100 µM) as additive, which itself alone does not show significant cytotoxicity towards AGS cells (see Fig. 3, negative control). Also, the peptide ligands **a**-**c**, **GGH** (262.5 µM) are not cytotoxic or at least only slightly cytotoxic, respectively (cell viability > 85%), independent on the usage of AscH^−^ as additive.

In contrast, a treatment with complexes **1**–**3**, **Cu-GGH** and the metal salt CuCl_2_ alone (all 250 µM) led to a decrease in cell viability (ranging from 79 to 9%). Thereby, in absence of AscH^−^ the same trend in cytotoxicity was recently observed by us: Gly2 (**1**, **Cu-GGH**) < CuCl_2_ ≤ β-Ala2 (**2**, **3**) [18]. Notably in this work, the cytotoxic effect of all complexes is enhanced by adding AscH^−^ as additive (without AscH^−^: ranging from 79 to 40% → with AscH^−^: ranging from 64 to 9%). Such an increase in activity by addition of AscH^−^ was also observed by Pauling et al*.* by the Gly2 complex **Cu-GGH** on Ehrlich ascites tumor cells [10]. Here, this cytotoxicity increase occurs also with the complexes **1**–**3,** which bear a Ser-Ser-tail at the *C*-terminus. Remarkably, the enhanced cytotoxic effect by adding AscH^−^ is up to three times higher on β-Ala2 complexes **3** (60 → 14%) and **2** (40 → 11%) in comparison to Gly2 complex **1** (80 → 64%). This is in accordance with our previous findings, that in the presence of AscH^−^ β-Ala2 complexes **2** and **3** are significantly more active in in vitro biological assays (ROS generation, DNA cleavage) when compared to their Gly2 analogs [18]. CuCl_2_ and β-Ala2 complexes **2** and **3**, respectively, show similar cytotoxic activities in the presence and absence of AscH^−^, which can be explained by their similar partition coefficients log *P* (vide infra, in vitro lipophilicity experiment). As positive control NEM (30 µM) was used for efficient cancer cell death [24].

Cytotoxicity trends were similar in HeLa and NCI-N87 cells, although the effects in these two cell lines are lower (down to max 11% cell viability on HeLa and 62% on NCI-N87). Also, the impact of AscH^−^ on the cytotoxicity is not that prominent as in AGS cells (see Fig. S5 and S6). Indeed, a 100 µM treatment of the studied cancer cell lines (AGS, Hela, NCI-N87) in the absence or presence of AscH^−^ (100 µM) led to a decrease in the cytotoxic effect of all compounds, but with the same trends as for a 250 µM treatment. Additionally, the NCI-N87 cells are more resistant towards a 100 µM compound treatment than AGS and HeLa cells (see Fig. S7–S9).

**Fig. 4:**
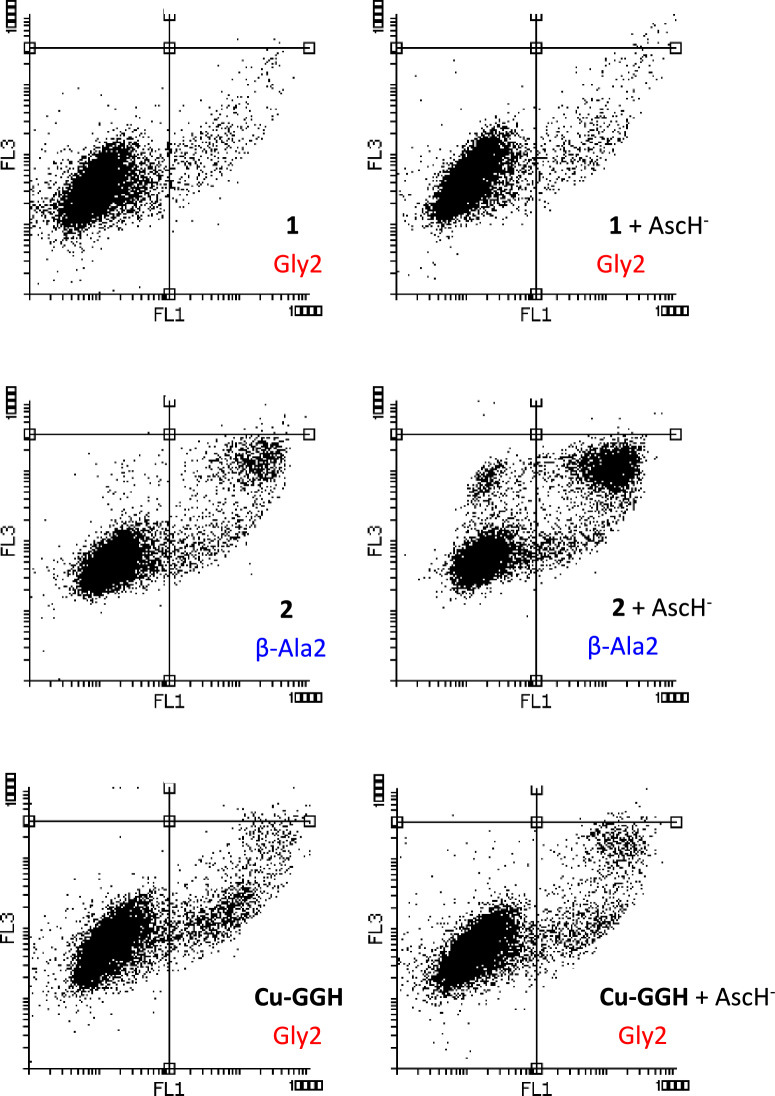
Fluorescence plots from the annexin V-FITC apoptosis assay of **1**, **2** and **Cu-GGH** (500 µM) in the absence and presence of AscH^−^ (100 µM) as additive on AGS cells. Treatment time with compound was 40 h. In the plots, cell counting in the left lower corner correspond to non-apoptotic cells (no fluorescence), whereas counting in the right lower corner to early apoptotic cells (green fluorescence) and right upper corner to late apoptotic/necrotic cells (mixed green–red fluorescence). X-axis relates to green (annexin V-FITC) and y-axis to red fluorescence (PI)

To corroborate the cell viability results of all compounds towards AGS, NCI-N87 and HeLa cells via the MTT assay, additionally the annexin V-FITC apoptosis assay was carried out under the same conditions, except for the incubation time (40 h) and the treatment concentration (500 µM). Through this assay it can be distinguished if a (cytotoxic) compound led to an early or late apoptotic, or even necrotic stage, and therefore allows to follow the cell death [25]. The fluorescent-protein-conjugate annexin V-FITC binds to phosphatidylserine (PS), an inner membrane phospholipid exposed to the cytoplasm in non-apoptotic cells, only if it is internalized into the outer cell membrane bilayer, which eventually occurs during apoptosis process (*e.g.* induced by cytotoxic metallopeptides). After binding, it shows green fluorescence (λ_em_ ≈ 530 nm) when it is light stimulated [26]. Another reagent used in the assay is DNA-intercalative but not cell-penetrating PI. If the cell membrane morphology/integrity is lost during (late stage) apoptosis, PI can bind to the DNA, and hence succeed in red fluorescence (λ_em_ > 600 nm) after light stimulus [27]. Thus, either green (PS binding) or a green–red mixture (PS and PI binding) is observed by the FACS technique via a flow cytometer, early or late apoptotic/necrotic events on cell death can be detected [25]. Exemplarily, in Fig. 4, the fluorescence plots resulting from the annexin V-FITC apoptosis assay (40 h compound treatment) on AGS cells are shown for the Gly2 complexes **1** and **Cu-GGH**, the β-Ala2 complex **2** (all 500 µM) in absence and presence of AscH^−^ (100 µM) (for **3**, CuCl_2_ and **a**, as exemplary peptide ligand, and the control (no compound treatment) see Fig. S10 and S11, and for HeLa and NCI-N87 cells see Fig. S12-S15, for quantitative apoptosis data on AGS, HeLa and NCI-N87 cells see Fig. S16-S18). A complex concentration of 500 µM was used due to a higher amount of cells (250 000 *vs*. 10 000 cells in the MTT assay).

Overall, on AGS cells, the annexin V-FITC apoptosis assay provides the same biological activity correlation of all compounds (complexes, peptide ligands, CuCl_2_) as the MTT assay supporting the found cytotoxicity trend: Gly2 complexes **1** and **Cu-GGH** < β-Ala2 complexes **2** and **3** ≈ CuCl_2_ (*cf.* Figure 4, S10 and S11). Whereas the cytotoxic effect of **1** and **Cu-GGH** without AscH^−^ led the cancer cells just to an early apoptotic stage in a moderate amount (< 9% apoptosis, see Fig. S16), which is not significantly affected by the addition of AscH^−^ (< 10%, see Fig. S16) (see Fig. 4, top and below), β-Ala2 complex **2** (and **3**, see Fig. S11) caused this AGS cell death stage to a higher amount even without AscH^−^ (15% apoptosis, Fig. S16) (confirmed also by the MTT assay on AGS cells, see Fig. 3), which is one key finding of this work. Addition of AscH^−^ to **2**, led to a remarkable increase in cytotoxicity bringing significantly more cells to a late apoptotic, or even necrotic stage (28% apoptosis, Fig. S16) (see Fig. 4, middle). Thus, a usage of AscH^−^ as additive has an unprecedented greater impact on the cytotoxic effect of β-Ala2 complexes **2** and **3** (see Fig. S10), than in comparison to **Cu-GGH** from Linus Pauling et al. [10]. The complex **Cu-GGH** was investigated over decades regarding its structure-biological activity relationship [1, 5, 12, 13], but only small improvements were reached through modification of **Cu-GGH** [14, 15]. Only targeting groups, such as acridine for G-quadruplex DNA targeting, led to an improvement of **Cu-GGH** through an adduct formation [28]. Here, even without applying targeting strategies by bioconjugation with the ATCUN peptide, increased cytotoxicity was observed. Cytotoxicity of β-Ala2 ATCUN complexes was enhanced by using AscH^−^ as a key additive. This finding represents the second main outcome of this work. Indeed, as in the MTT assay also on HeLa and NCI-N87 cells, the same activity trend of all compounds could be observed regarding the annexin V-FITC apoptosis assay, again with slightly lower activities (see Fig. S12-S15). However, on HeLa cells in the annexin V-FITC apoptosis assay especially the β-Ala2 complexes and CuCl_2_ cause more apoptosis than in the MTT assay. Nevertheless, the impact of AscH^−^ is similar in both assays on all three cell lines, *i.e.* is greater on AGS cells (*cf.* Figure 4, S5-S6 with S16-S18).

In all three cancer cell lines, the copper salt CuCl_2_ shows a cytotoxic effect in the same range as β-Ala2 complexes **2** and **3** (*cf.* Figures 3, 4 and S10). To describe this phenomenon, we carried out the *n*-octanol/water experiment, which delivers information about the lipophilicity (log *P* values), and hence differences in the strength of cell permeability. In Table 1, the log *P* values are listed for the complexes **1**–**3**, **Cu-GGH** and CuCl_2_.

**Table 1:** In vitro lipophilicity via* n*-octanol–water partition coefficients (log *P* values) of Cu(II) complexes and CuCl_2_

Compound	log *P*
**1**	− 0.98
**2**	− 0.75
**3**	− 0.82
**Cu-GGH**	− 0.75
CuCl_2_	− 0.87

The lipophilicity properties of all complexes and CuCl_2_ are in the same order of magnitude. However, since **2** and **3** have a slightly higher lipophilicity (less negative log *P* values) than CuCl_2_ (see Table 1), it is likely that these complexes can more easily penetrate the cell membranes under in vitro cell culture conditions. This may result in a higher content of intracellular cytotoxic Cu(II) in the studied cancer cells for **2** and **3** than for CuCl_2_. It is known that the cytotoxicity of copper is shielded by intracellular metallothioneins binding [29]. ATCUN-like peptide ligands could act in a same way for attenuating the cytotoxicity. This may result in balancing of both effects (cytotoxicity of bound/unbound copper *vs.* cell permeability) yielding nearly same antiproliferative activities for both, β-Ala2 complexes **2** and **3** and CuCl_2_. Additionally, since the complex stability of β-Ala2 complexes **2** and **3** are lower than its Gly2 analogs [18], it also reasonable that the β-Ala2 complexes dissociate intracellularly more likely, which causes comparable cytotoxic effects for **2**, **3** and CuCl_2_. This phenomenon is already known for Cu(II) phenanthroline complexes [30].

It is well known, that Cu(II) is toxic to cells in general (independently on cancer or non-tumorigenic cells) [31], but due to a lack of selectivity towards cancer cells it is not meaningful to inject a Cu(II) salt into the human body as a chemotherapeutic agent. Thus, by using peptide ligands, such as β-Ala2 ATCUN motifs, for complexing cytotoxic Cu(II) has biorelevant advantages in two different ways: On one hand, the high cytotoxicity of “free” Cu(II) is attenuated by its peptide complex, which is in accordance with the in vitro lipophilicity/cytotoxicity correlation (vide supra). On the other hand, the peptide ligand delivers a scaffold for potential conjugation with (peptidic) tumor targeting groups [28, 32] to address cancer cells (*e.g.* overexpressed receptors on tumor cells [33]). Notably, β-Ala2 complexes **2** and **3** are more bioactive than Gly2 complexes **1** and **Cu-GGH**. Moreover, **2** and **3** show cytotoxic activities in the same order of magnitude as CuCl_2_ on the tested cancer cell lines. Nevertheless, peptide scaffolds **b** and **c** in comparison to “free” Cu(II) exhibits potential in designing of drug delivery systems for cytotoxic Cu(II).

## Conclusion

Cu(II) ATCUN peptide complexes are under biological studies since Linus Pauling et al. observed an antitumor activity of Cu-GGH in 1983, which is even higher in the presence of AscH^−^ as additive [10]. However, over the decades, no significant improvements in biological activities of complexes of the type **Cu(II)-GGH** were achieved (not considering targeting strategies) and also the usage of AscH^−^ has fallen into oblivion.

Here, we studied in vitro physiologically stable metallopeptides regarding their cytotoxicity towards AGS, HeLa and NCI-N87 cancer cells via the MTT and the annexin V-FITC apoptosis (flow cytometry) assays. It could be shown, that in absence of AscH^−^ as additive Gly2 complexes **1** and **Cu-GGH** are less active than β-Ala2 complexes **2** and **3** on the tested cancer cell lines. In contrast, in the presence of AscH^−^ the antiproliferative activity of Gly2 complexes **1** and **Cu-GGH** is moderately increased, which is accordance with the findings of Linus Pauling et al. [10]. Remarkably in this work, a cytotoxicity enhancement by adding AscH^−^ is up to three-fold higher on β-Ala2 complexes **2** and **3** than on Gly2 complexes **1** and **Cu-GGH.** This is in line with previous observations in in vitro bioanalytical experiments when adding AscH^−^, where the Gly2 complexes were less active than the β-Ala2 complexes, e.g. in DNA cleavage experiments [18].

Notably, in the apoptosis assay CuCl_2_ alone exhibits an antiproliferative activities in the same order of magnitude as β-Ala2 complexes **2** and **3**. We assume a balancing effect between cell permeability and cytotoxic effect of bound Cu(II) (peptidic complexes)/unbound Cu(II) (CuCl_2_) under in vitro experimental conditions. It is likely that the bound Cu(II) in **2** and **3** is less toxic, but possesses a higher cell permeability, which is in accordance with their lipophilicity properties (log *P* values).

The findings of this work, could lead to a renaissance of AscH^−^ usage after its oblivion on Cu(II) ATCUN peptide complexes, especially with β-Ala-type peptides, in antitumor activity investigations and brings the compound class metallopeptides a step forward as a chemotherapeutic agent.

## Supplementary Information

Below is the link to the electronic supplementary material.

Supplementary file1 (DOCX 400 KB)

## Data Availability

No datasets were generated or analysed during the current study.

## Abbreviations

β-Ala2: β-Alanine in 2nd position of the peptide sequence from the N-terminus
AscH^−^: Ascorbate
ATCUN: Amino terminal Cu(II) and Ni(II) binding
DNA: Deoxyribonucleic acid
FACS: Fluorescence-assisted cell sorting
FITC: Fluorescein isothiocyanate
GGH: NH_2_-Gly-Gly-His-COOH
Gly2: Glycine in 2nd position of the peptide sequence from the N-terminus
MTT: 3-(4,5-Dimethylthiazol-2-yl)-2,5-diphenyltetrazolium bromide
NEM: *N*-Ethylmaleimide
PI: Propidium iodide
PS: Phosphatidylserine
ROS: Reactive oxygen species
