# Supplementary material for: Ascorbate: a forgotten component in the cytotoxicity of Cu(II) ATCUN peptide complexes
Source: J Biol Inorg Chem. 2024 Nov 11;29(7-8):801–9. doi: 10.1007/s00775-024-02083-9 (PMC11638278; doi:10.1007/s00775-024-02083-9)
Supplement: Supplementary file 1 — Supplementary file1 (DOCX 400 KB) [file 775_2024_2083_MOESM1_ESM.docx]

**Supplementary information**

**Ascorbate: a forgotten component in the cytotoxicity of Cu(II) ATCUN peptide complexes**

Julian Heinrich^1,2^, Elisa Siddiqui^3^, Henrike Eckstein^3^, Michael Naumann^3 *^, Nora Kulak^1,2 *^

^1^ Institute of Chemistry, University of Potsdam, Karl-Liebknecht-Straße 24-25, 14476 Potsdam, Germany

^2^ Institute of Chemistry, Otto von Guericke University, Universitätsplatz 2, 39106 Magdeburg, Germany

^3^ Institute of Experimental Internal Medicine, Otto von Guericke University, Leipziger Straße 44, 39120 Magdeburg, Germany

**Table of contents**

1. **UV/VIS spectroscopy**
2. **Cytotoxicity** **in AGS, HeLa and NCI-N87 cancer cells**
   1. **MTT cell viability assay**
   2. **Annexin V-FITC apoptosis assay**
3. **UV/VIS spectroscopy**

|  |
| --- |
| **Fig. S1** UV/VIS spectra in the range 400–700 nm of 1 mM **1** (Cu(II):peptide ratio = 1:1.05) in 50 mM HEPES (pH 7.4) at 37 °C recorded at different time points (0 h 🡪 48 h). Time-dependent changes in the d-d transition band of **1** at λ_max_ = 525 nm were utilized to evaluate the complex stability at physiological conditions. |

|  |
| --- |
| **Fig. S2** UV/VIS spectra in the range 400–700 nm of 1 mM **2** (Cu(II):peptide ratio = 1:1.05) in 50 mM HEPES (pH 7.4) at 37 °C recorded at different time points (0 h 🡪 48 h). Time-dependent changes in the d-d transition band of **2** at λ_max_ = 545 nm were utilized to evaluate the complex stability at physiological conditions. |

In the UV/VIS complex stability experiment for **2** (Fig. S2) another absorption band in the region <400 nm builds up over 48 h, however, the d-d transition band is not significantly affected by this new band emergence. Peptide **c** (complex **3**) bears two Lys residues in position 1 and 4 from the *N*-terminus of the amino acid sequence in comparison to peptide **b** (complex **2**), which is the only structural difference in **3** *vs.* **2** (notable, both complexes comprise a Ser-Ser-tail at the *C*-terminus and a β-Ala in position 2 of the sequence (Fig. 1)). It is known, that Cu(II) complexes, such as **2**, tend to second-sphere Cu(II) coordination by a hydroxyl group of the Ser-Ser-tail, which is not the case for **3** [1]. Furthermore, the amino acid Ser shows a broad absorption band in the region <375 nm and a sharp and strong band around 210 nm [2]. Thus, we assume that in **2** the band occurrence <400 nm is caused by second-sphere interactions of the Ser-Ser-tail with already 4N-complexed Cu(II). In **3** the two bulky Lys residues pretend the Cu(II) center from Ser-second-sphere interaction [1], since this band occurrence <400 nm is not observed for **3** (Fig. S3). Nevertheless, for this work the stability of the d-d transition band of **2** is crucial for evaluation of the complex stability in *in vitro* cell culture experiments, which stays unaffected. Consequently, the complex stability of **2** and therefore its bioactivity remains in the corresponding time range of cytotoxicity experiments.

Complex **1** is also bearing the Ser-Ser tail, however a band emergence <400 nm does not occur. This indicates that second-sphere interaction is not of relevance for the GGH-based complex with its more strongly binding GGH chelate (5,5,6) [1].

|  |
| --- |
| **Fig. S3** UV/VIS spectra in the range 400–700 nm of 1 mM **3** (Cu(II):peptide ratio = 1:1.05) in 50 mM HEPES (pH 7.4) at 37 °C recorded at different time points (0 h 🡪 48 h). Time-dependent changes in the d-d transition band of **3** at λ_max_ = 545 nm were utilized to evaluate the complex stability at physiological conditions. |

|  |
| --- |
| **Fig. S4** UV/VIS spectra in the range 400–700 nm of 1 mM **Cu-GGH** (Cu(II):peptide ratio = 1:1.05) in 50 mM HEPES (pH 7.4) at 37 °C recorded at different time points (0 h 🡪 48 h). Time-dependent changes in the d-d transition band of **Cu-GGH** at λ_max_ = 525 nm were utilized to evaluate the complex stability at physiological conditions. |

1. **Cytotoxicity on AGS, HeLa and NCI-N87 cancer cells**
   1. **MTT cell viability assay**

|  |
| --- |
| **Fig. S5** Cytotoxic effect of the complexes **1**-**3**, **Cu-GGH** (250 µM) and their corresponding peptide ligands **a**-**c**, **GGH** alone (262.5 µM) and CuCl_2_, indicated as Cu(II), (250 µM) on the cell viability of HeLa cancer cells in the absence/presence (100 µM) of AscH^-^ as additive measured by the MTT assay after 48 h treatment. Higher concentrations of the ligands were used in accordance to the Cu(II):peptide ratio in the complexes of 1:1.05. |

|  |
| --- |
| **Fig. S6** Cytotoxic effect of the complexes **1**-**3**, **Cu-GGH** (250 µM) and their corresponding peptide ligands **a**-**c**, **GGH** alone (262.5 µM) and CuCl_2_, indicated as Cu(II), (250 µM) on the cell viability of NCI-N87 cancer cells in the absence/presence (100 µM) of AscH^-^ as additive measured by the MTT assay after 48 h treatment. Higher concentrations of the ligands were used in accordance to the Cu(II):peptide ratio in the complexes of 1:1.05. |

|  |
| --- |
| **Fig. S7** Cytotoxic effect of the complexes **1**-**3**, **Cu-GGH** (100 µM) and their corresponding peptide ligands **a**-**c**, **GGH** alone (105 µM) and CuCl_2_, indicated as Cu(II), (100 µM) on the cell viability of AGS cancer cells in the absence/presence (100 µM) of AscH^-^ as additive measured by the MTT assay after 48 h treatment. Higher concentrations of the ligands were used in accordance to the Cu(II):peptide ratio in the complexes of 1:1.05. |

|  |
| --- |
| **Fig. S8** Cytotoxic effect of the complexes **1**-**3**, **Cu-GGH** (100 µM) and their corresponding peptide ligands **a**-**c**, **GGH** alone (105 µM) and CuCl_2_, indicated as Cu(II), (100 µM) on the cell viability of HeLa cancer cells in the absence/presence (100 µM) of AscH^-^ as additive measured by the MTT assay after 48 h treatment. Higher concentrations of the ligands were used in accordance to the Cu(II):peptide ratio in the complexes of 1:1.05. |

|  |
| --- |
| **Fig. S9** Cytotoxic effect of the complexes **1**-**3**, **Cu-GGH** (100 µM) and their corresponding peptide ligands **a**-**c**, **GGH** alone (105 µM) and CuCl_2_, indicated as Cu(II), (100 µM) on the cell viability of NCI-N87 cancer cells in the absence/presence (100 µM) of AscH^-^ as additive measured by the MTT assay after 48 h treatment. Higher concentrations of the ligands were used in accordance to the Cu(II):peptide ratio in the complexes of 1:1.05. |

- 1. **Annexin V-FITC apoptosis assay**

| 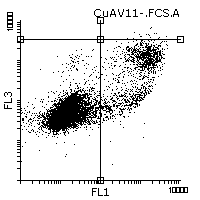 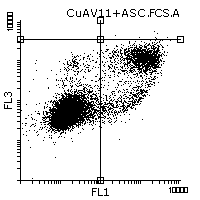  **3** + AscH^-^  β-Ala2  **3**  β-Ala2        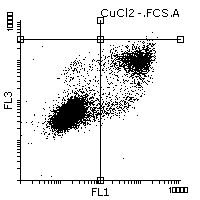 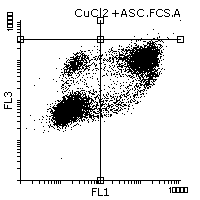  Cu(II)  Cu(II) + AscH^-^ |
| --- |
| **Fig. S10** Fluorescence plots from the annexin V-FITC apoptosis assay of **3**, CuCl_2_ (both 500 µM) in the absence and presence of AscH^-^ (100 µM) as additive on AGS cells. Treatment time with compound was 40 h. In the plots, cell counting in the left lower corner correspond to healthy cells (no fluorescence), whereas counting in the right lower corner to early apoptotic cells (green fluorescence) and right upper corner to late apoptotic/necrotic cells (mixed green-red fluorescence). X-axis relates to green (annexin V-FITC) and y-axis to red fluorescence (PI). |

| 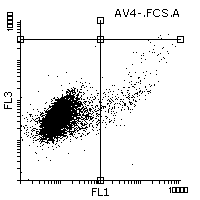 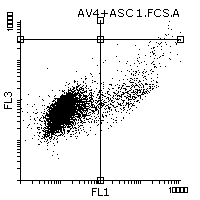  **a** + AscH^-^  **a**    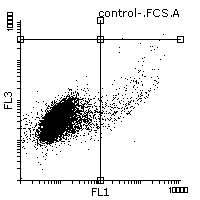  control |
| --- |
| **Fig. S11** Fluorescence plots from the annexin V-FITC apoptosis assay of **a** (525 µM) in the absence and presence of AscH^-^ (100 µM) as additive on AGS cells. The control experiment contains no compound. Treatment time with or without compound was 40 h. In the plots, cell counting in the left lower corner correspond to healthy cells (no fluorescence), whereas counting in the right lower corner to early apoptotic cells (green fluorescence) and right upper corner to late apoptotic/necrotic cells (mixed green-red fluorescence). X-axis relates to green (annexin V-FITC) and y-axis to red fluorescence (PI). |

| 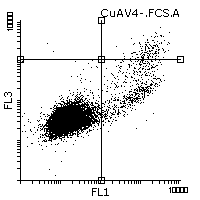 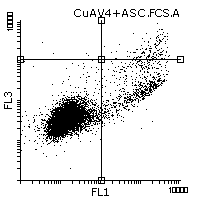  **1** + AscH^-^  Gly2  **1**  Gly2      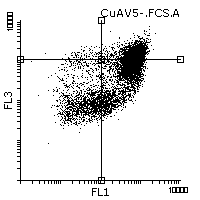 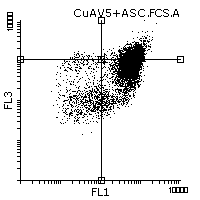  **2** + AscH^-^  β-Ala2  **2**  β-Ala2    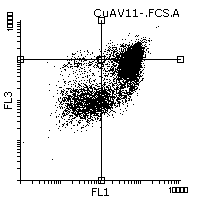 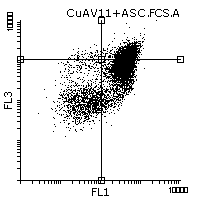    **3** + AscH^-^  β-Ala2  **3**  β-Ala2    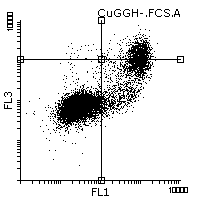 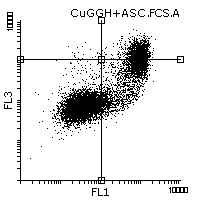    **Cu-GGH** + AscH^-^  Gly2  **Cu-GGH**  Gly2 |
| --- |
| **Fig. S12** Fluorescence plots from the annexin V-FITC apoptosis assay of **1**, **2**, **3** and **Cu-GGH** (all 500 µM) in the absence and presence of AscH^-^ (100 µM) as additive on HeLa cells. Treatment time with compound was 40 h. In the plots, cell counting in the left lower corner correspond to healthy cells (no fluorescence), whereas counting in the right lower corner to early apoptotic cells (green fluorescence) and right upper corner to late apoptotic/necrotic cells (mixed green-red fluorescence). X-axis relates to green (annexin V-FITC) and y-axis to red fluorescence (PI). |

| 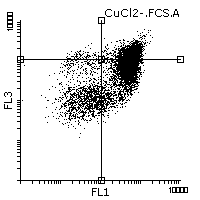 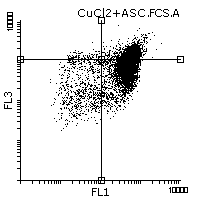  Cu(II)  Cu(II) + AscH^-^      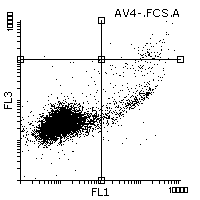 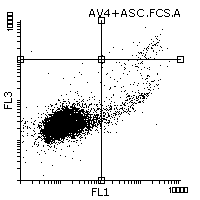  **a** + AscH^-^  **a**    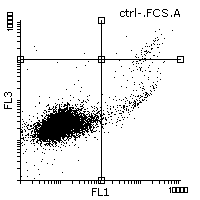    control |
| --- |
| **Fig. S13** Fluorescence plots from the annexin V-FITC apoptosis assay of CuCl_2_ (500 µM), **a** (525 µM) in the absence and presence of AscH^-^ (100 µM) as additive on HeLa cells. The control experiment contains no compound. Treatment time with compound was 40 h. In the plots, cell counting in the left lower corner correspond to healthy cells (no fluorescence), whereas counting in the right lower corner to early apoptotic cells (green fluorescence) and right upper corner to late apoptotic/necrotic cells (mixed green-red fluorescence). X-axis relates to green (annexin V-FITC) and y-axis to red fluorescence (PI). |

| 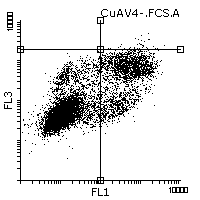 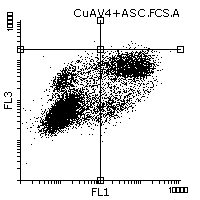  **1** + AscH^-^  Gly2  **1**  Gly2      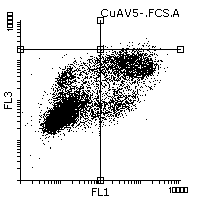 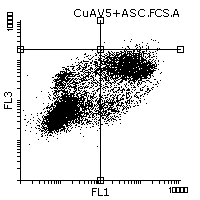  **2** + AscH^-^  β-Ala2  **2**  β-Ala2    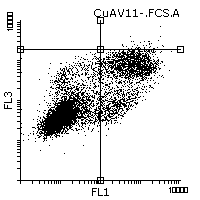 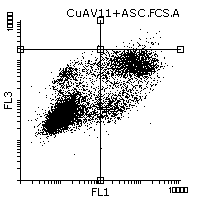    **3** + AscH^-^  β-Ala2  **3**  β-Ala2    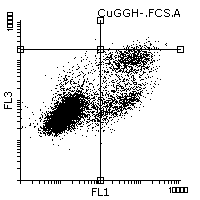 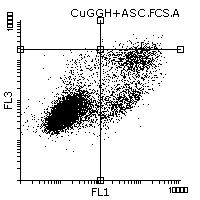  **Cu-GGH**  Gly2    **Cu-GGH** + AscH^-^  Gly2 |
| --- |
| **Fig. S14** Fluorescence plots from the annexin V-FITC apoptosis assay of **1**, **2**, **3** and **Cu-GGH** (all 500 µM) in the absence and presence of AscH^-^ (100 µM) as additive on NCI-N87 cells. Treatment time with compound was 40 h. In the plots, cell counting in the left lower corner correspond to healthy cells (no fluorescence), whereas counting in the right lower corner to early apoptotic cells (green fluorescence) and right upper corner to late apoptotic/necrotic cells (mixed green-red fluorescence). X-axis relates to green (annexin V-FITC) and y-axis to red fluorescence (PI). |

| 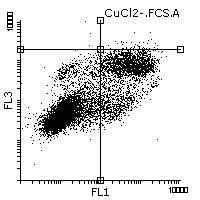 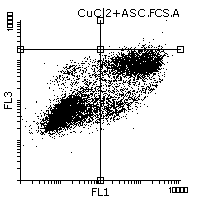  Cu(II)  Cu(II) + AscH^-^      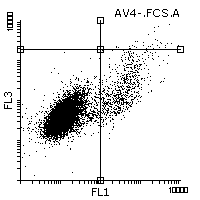 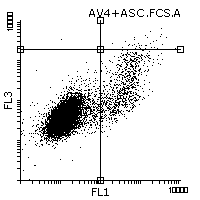  **a** + AscH^-^  **a**    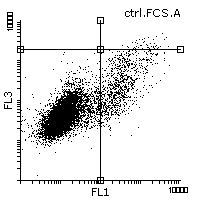    control |
| --- |
| **Fig. S15** Fluorescence plots from the annexin V-FITC apoptosis assay of CuCl_2_ (500 µM), **a** (525 µM) in the absence and presence of AscH^-^ (100 µM) as additive on NCI-N87 cells. The control experiment contains no compound. Treatment time with compound was 40 h. In the plots, cell counting in the left lower corner correspond to healthy cells (no fluorescence), whereas counting in the right lower corner to early apoptotic cells (green fluorescence) and right upper corner to late apoptotic/necrotic cells (mixed green-red fluorescence). X-axis relates to green (annexin V-FITC) and y-axis to red fluorescence (PI). |

|  |
| --- |
| **Fig. S16** Apoptosis in percentage induced by the complexes **1**, **2**, **3**, **Cu-GGH** (500 µM) and **a**, as exemplary peptide ligand (525 µM) and CuCl_2_, indicated as Cu(II) (500 µM) in the absence and presence of AscH^-^ (100 µM) on AGS cells followed by the annexin V-FITC apoptosis assay (40 h compound incubation time). |

|  |
| --- |
| **Fig. S17** Apoptosis in percentage induced by the complexes **1**, **2**, **3**, **Cu-GGH** (500 µM) and **a**, as exemplary peptide ligand (525 µM) and CuCl_2_, indicated as Cu(II) (500 µM) in the absence and presence of AscH^-^ (100 µM) on HeLa cells followed by the annexin V-FITC apoptosis assay (40 h compound incubation time). |

|  |
| --- |
| **Fig. S18** Apoptosis in percentage induced by the complexes **1**, **2**, **3**, **Cu-GGH** (500 µM) and **a**, as exemplary peptide ligand (525 µM) and CuCl_2_, indicated as Cu(II) (500 µM) in the absence and presence of AscH^-^ (100 µM) on NCI-N87 cells followed by the annexin V-FITC apoptosis assay (40 h compound incubation time). |

**References**

1. Heinrich J, Bossak-Ahmad K, Riisom M, Haeri HH, Steel TR, Hergl V, Langhans A, Schattschneider C, Barrera J, Jamieson SMF, Stein M, Hinderberger D, Hartinger CG, Bal W, Kulak N (2021) Chem Eur J 27:18093–18102.
2. Rajesh K, Praveen Kumar P (2014) J Mater 2014:790957.
